# Generating high-fidelity synthetic patient data for assessing machine learning healthcare software

**DOI:** 10.1038/s41746-020-00353-9

**Published:** 2020-11-09

**Authors:** Allan Tucker, Zhenchen Wang, Ylenia Rotalinti, Puja Myles

**Affiliations:** 1 Department of Computer Science, Brunel University London, London, UK; 2 CPRD, Medicines & Healthcare Products Regulatory Agency, London, UK; 3 Biomedical Informatics Laboratory, University of Pavia, Pavia, Italy

**Keywords:** Epidemiology, Statistics, Computer science, Computational science, Risk factors

## Abstract

There is a growing demand for the uptake of modern artificial intelligence technologies within healthcare systems. Many of these technologies exploit historical patient health data to build powerful predictive models that can be used to improve diagnosis and understanding of disease. However, there are many issues concerning patient privacy that need to be accounted for in order to enable this data to be better harnessed by all sectors. One approach that could offer a method of circumventing privacy issues is the creation of realistic synthetic data sets that capture as many of the complexities of the original data set (distributions, non-linear relationships, and noise) but that does not actually include any real patient data. While previous research has explored models for generating synthetic data sets, here we explore the integration of resampling, probabilistic graphical modelling, latent variable identification, and outlier analysis for producing realistic synthetic data based on UK primary care patient data. In particular, we focus on handling missingness, complex interactions between variables, and the resulting sensitivity analysis statistics from machine learning classifiers, while quantifying the risks of patient re-identification from synthetic datapoints. We show that, through our approach of integrating outlier analysis with graphical modelling and resampling, we can achieve synthetic data sets that are not significantly different from original ground truth data in terms of feature distributions, feature dependencies, and sensitivity analysis statistics when inferring machine learning classifiers. What is more, the risk of generating synthetic data that is identical or very similar to real patients is shown to be low.

## Introduction

It is increasingly evident that the use of historical data within health systems can offer huge rewards in terms of increased accuracy, timely diagnoses, the discovery of new knowledge about disease and its progression, and the ability to offer a more personalised prognosis and care pathway for patients^1^. What is more, there is a huge demand from the public and governments to make new technology available within health services as quickly as possible while ensuring that any software that uses Artificial Intelligence (AI), in particular Machine Learning, is robustly validated to check for biases and errors^2^.

Many issues concerning patient privacy have been highlighted since the introduction of General Data Protection Regulation^3^. This includes protections from the identification of an individual’s data within large data samples^4^ and the right to explanation for any decision that is made by an automated system^5^. As a result of this legislation, the ability to offer large samples of real individual-level patient data to companies and institutions is limited. One possible solution to this problem is the use of synthetic data as an alternative to assist in the rapid development and validation of new tools. This data must capture all of the correct (potentially non-linear and multivariate) dependencies and distributions that are apparent in the real data sets, while also preserving patient privacy and avoiding the risks of individual identification.

In this paper, we explore some of the key issues in generating realistic and useful synthetic data, namely preserving relationships, distributions, predictive capabilities, and patients’ privacy. We also explore what robust methods need to be used to validate models using synthetic data in order to ensure biases in the models, overfitting issues, and high variance are discovered and reported. The paper is broken down in to three main sections: first, we discuss some of the key issues concerning the generation and use of synthetic data and introduce a method based on probabilistic graphical models; second, we explore a case study using primary care data from the Clinical Practice Research Datalink (CPRD) in the UK. CPRD is a real-world research service supporting retrospective and prospective public health and clinical studies. It is jointly sponsored by the Medicines and Healthcare products Regulatory Agency and the National Institute for Health Research, as part of the Department of Health and Social Care^6^. Finally, we make conclusions and recommendations about the advantages and disadvantages of using synthetic data for rapid development of AI systems in healthcare.

There are already existing methods for generating synthetic data. One simple approach is through data perturbation by adding noise to the original data set. For example, rotations, cropping, and noise injection in images^7–9^ in order to produce more diverse data sets for a more generalisable classifier, or through the addition of noise from some distribution such as the Laplace mechanism as used in PrivBayes^10^ in order to make it more difficult to identify individuals from a data set. Another approach uses *generative models* of data^11^. In this case, models that capture the correct relationships and distributions are built, either hand-coded based upon expert knowledge or inferred from real data using models such as Bayesian networks (BNs)^10,12^ or neural networks^13^. These can then be used to generate synthetic data via sampling techniques. Generative Adversarial Networks have become particularly popular as a method to generate synthetic image data to build more robust models containing fewer biases than those generated on real data alone^14^.

Bias in the data can appear due to the way data is collected. In many fields, data analysis involves using historical secondary-use data that was not collected for the analysis in question, as opposed to well-designed research data aimed at answering a specific statistical question (as found in clinical trials for example). This means that secondary-use data sets are often *imbalanced*, particularly in medicine. For example, in primary care data the number of patients with a specific disease may be far lower than patients who do not have the disease. Conversely, data that is collected by a particular hospital may not reflect the general population as less-severe patients may be managed in primary care, while the data collected in hospitals will only contain more severe patients who are already diagnosed with a specific disease or are at high risk of developing it. As a result, any models that are inferred from such data must deal with these imbalances, either through resampling methods^15,16^ or synthetic data generation. SMOTE is a commonly used resampling technique in machine learning for dealing with small and imbalanced samples and involves generating synthetic datapoints to supplement existing data^17^.

An important issue concerning the use of an underlying model to generate synthetic data is that the inherent biases may not be visible. For example, Neural Network approaches whereby models are inferred from data have turned out to be biased, leading to decisions and classifications being made for the wrong reasons^18^. Agnostic network approaches have attempted to deal with unwanted biases in the data by selecting known “protected concepts” and using domain adversarial training^19^ to account for these biases. The issue of bias is especially a problem for models where the relationships between features are not explicitly represented because unwanted correlations cannot easily be identified. This is known as the *black box* problem where it is difficult to know how a model will behave when it has many complex parameters that are not easily interpreted. Approaches that try to deal with this by modelling influences more transparently include probabilistic graphical models^20^ and tree-based models^21,22^.

Many data sets will contain specific characteristics that must be taken into account when learning a model for synthetic data generation. For example, missing data are common in most medical data sets. These missing data can manifest for many different reasons but if the data are not recorded for some systematic reason then this must be accounted for in the modelling process. This is structurally missing data—also known as Missing Not At Random (MNAR) as opposed to Missing At Random (MAR). If MNAR is *non-ignorable*, then we must find a way to model these types of missingness. For example, in probabilistic graphical models, a discrete variable can include a “missing” state, while continuous value variables can include a binary node representing whether the variable measurement is missing or not^23^. However, for non-ignorable MNAR data we need to use robust methods^24^. This is because the pattern of missing data can often have value in itself and be exploited to assist in making predictions^23^. Other approaches include explicitly modelling these unmeasured effects as latent variables^25^, which we will explore in this paper.

Most data sets will contain unmeasured effects. That is, some underlying processes that have not been recorded in the data (perhaps because they were not considered important at the time of collection, or perhaps because they were not known at the time—e.g. a particular clinical test that has been introduced part way through the data collection process). These can be modelled using latent variable approaches that use methods such as the FCI algorithm^20^ to infer the location and the Expectation Maximisation algorithm^26^ to infer the parameters of these unmeasured variables. A key issue being explored in this paper is how synthetic data can be used while ensuring patient privacy. That is, the ability to use simulated patient data to build new models without giving away personal information. There are a number of concepts that attempt to measure how easy it is to identify a patient from their data. For example, *k*-anonymisation is a measure of the least number of individuals (*k*) in a data set who share the set of attributes that might become identifying for each individual^27^, while *ε*-differential privacy is a metric which enables data managers to only release aggregates of data that cannot be used to identify individuals^28^. Re-identification has proven to be problematic, for example, through “differentiation attack” where aggregated data are repeatedly requested for different subsets to enable the attacker to identify individual. This is a risk even when data have been anonymised^29^. For many individuals, aggregated data can preserve their privacy if data cannot be repeatedly requested as they cannot be identified from the summary statistics/distributions that are learnt from a large population. However, people who are considered *outliers*, for example, those who have rare disease or demographics may still be identified. As a result, outlier analysis^30^ needs to be incorporated. Simply removing these patients may be an option but this can sometimes mean missing out on important data that could be used to help future patients.

In summary, there have been numerous attempts to generate synthetic data for different reasons, including to deal with biased, imbalanced, and small sampled data. There is now a push to explore how synthetic data may enable researchers to build predictive models while preserving patient privacy. In this paper, we explore the integration of probabilistic graphical models with latent variables and resampling to simultaneously capture many features of real-world complex primary care data, including missing data, non-linear relationships, and uncertainty, while focussing on the importance of transparency of the modelling and data generation process. In the next section, we describe the methods that we have adopted to construct and robustly validate synthetic data samples. We also describe the primary care data in detail. We then carry out an empirical analysis on a subset of the primary care data with a focus on cardiovascular risk. This includes an evaluation of our probabilistic graphical model approach to handling missing data by comparing the synthetic data to original ground truth data in terms of distributional characteristics. We then explore how the synthetic data compare on machine learning classification tasks by comparing the sensitivity analyses on synthetic and ground truth data.

## Results

### Data and modelling

Our experiments make use of the CPRD Aurum data set. This includes patient Electronic Healthcare Records collected routinely from primary care practices using the EMIS® patient management software system. When a practice agrees to contribute their patient data to CPRD Aurum, CPRD receives a full historic collection of the coded part of the practice’s electronic health records, which includes data on deceased patients and those who have left the practice. The coded clinical record includes symptoms, diagnoses, prescriptions, immunisations, tests, lifestyle factors, and referrals recorded by the general practitioner (GP) or other practice staff but does not include free text medical notes^6^. The November 2019 release of the CPRD Aurum database included a total of 27.5 million patients (including deceased and transferred patients) from 1042 practices of whom 9.7 million were currently registered with a GP^6^.

We have chosen a generative approach to modelling the CPRD data where the focus is on a combination of machine learning that is augmented with expert knowledge. This is because we want to ensure that any biases that occur in the ground truth data are made explicit and can be dealt with at each stage of the data generation process. As a result, the underlying model must deal with all the potential uncertainty in the data while also modelling the distributions and relationships in as transparent a manner as possible. For this reason, we have chosen a BN framework. The first experiment involved learning a BN from the CPRD data set.

**Fig. 1:**
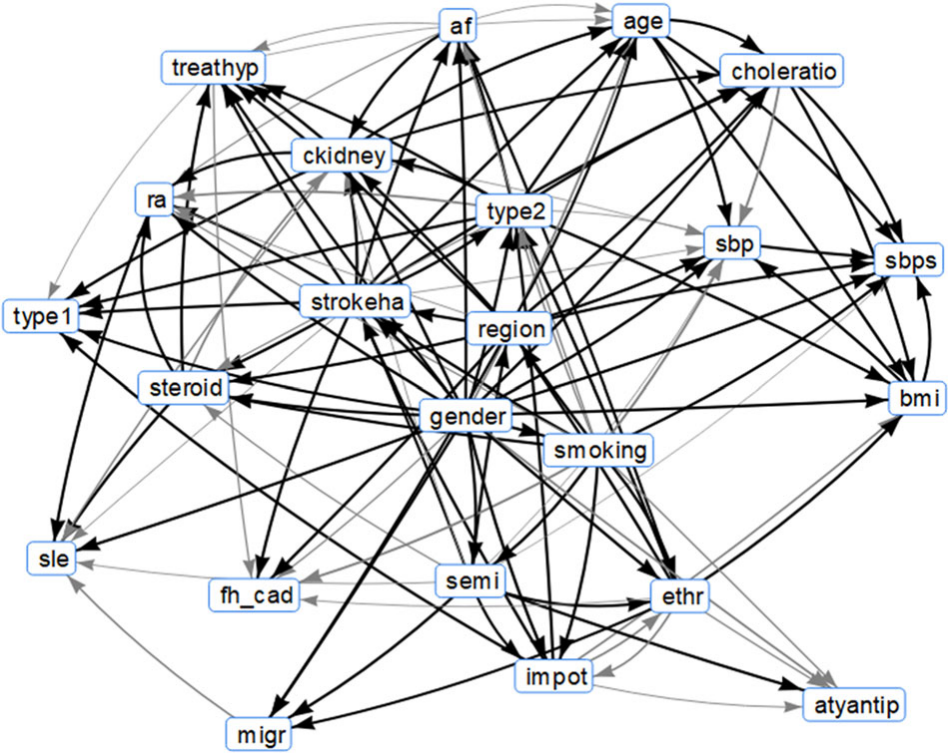
Resultant graph structure for BNs learnt from samples of ground truth data. Confidences of 100% are represented by black arcs while those <100% are represented by varying widths in grey.

The general structure of the discovered BNs from multiple samples of the original CPRD data, which we denote as ground truth (GT), are shown in Fig. 1. This explicit representation of independencies between variables allows experts to assess the underlying model and check for potential biases within the GT data. For example, almost all the black arc relationships are well recognised in medical research:

- Cholesterol/high-density lipoprotein ratio and type 2 diabetes: increased ratio in type 2 diabetes^31^
- Steroid treatment and systemic lupus erythematosus (SLE): steroids used in SLE treatment^32^
- Rheumatoid arthritis (RA) and SLE—both are autoimmune conditions, and while RA affects joints, SLE can affect joints in some variants and mimic RA. They are considered distinct diseases but can co-occur^33^
- Severe mental illness and migraines: migraines can precede mental illness and are common in those with anxiety disorders^34^
- Smoking and severe mental illness: well-known association, especially in schizophrenia (widely observed but may not be causal)^35^
- Ethnicity and body mass index (BMI): possibly confounded by lifestyle explanations but widely observed association^36^
- Smoking and systolic blood pressure: the grey in the network reflects the conflicting evidence base in this area^37^
- Smoking and impotence; this also explains why there is a relationship between the male gender and impotence^38^
- Type 1 diabetes and impotence^39^
- Age and systolic blood pressure: increasing systolic blood pressure with increasing age^40^
- Family history of coronary heart disease increases risk of stroke/heart attacks^41^
- Antipsychotics and severe mental illness: antipsychotics used for treatment of severe mental illness (bnf.nice.org.uk)
- Systolic blood pressure and systolic blood pressure SD: correlated variables
- Atrial fibrillation (AF) and stroke/heart attack: AF is risk factor for stroke (stroke.org.uk)
- Chronic kidney disease and stroke/heart attacks: often co-occur^42^
- Age and type 2 diabetes: increasing risk of type 2 diabetes with age^39^

There were, however, some surprises:

- Region was connected to impotence. Perhaps there is an indirect link as linked to regional distribution of smoking^43^
- There is no clear link between systolic blood pressure and blood pressure treatment. This could possibly be due to systolic blood pressure being a numeric variable spanning normal and high systolic blood pressure readings

We adopt three BN modelling approaches to handle missing data: First, we simply delete all cases with missing data. Second, we model missingness in discrete nodes by adding a “Miss State” to all possible node states, and in continuous nodes by adding a new binary parent (a “Miss Node”) to each node, representing whether the data point is missing or not. Finally, we explore the use of the FCI algorithm^20^ to infer any latent variables in the network. These methods are explained in more detail in the “Methods” section. The following links to 6 latent variables were discovered:

“L1” → “age”

“L1” → “af”

“L1” → “treathyp”

“L2” → “steroid”

“L2” → “treathyp”

“L3” → “impot”

“L3” → “gender”

“L4” → “migr”

“L4” → “choleratio”

“L4” → “gender”

“L5” → “strokeha”

“L5” → “ckidney”

“L5” → “type2”

“L5” → “choleratio”

“L5” → “sbps”

“L6” → “strokeha”

“L6” → “ckidney”

“L6” → “type2”

Having accepted this underlying BN model (though we can choose to update it based on expert knowledge by removing known false links and adding expected true links), we now explore how it can generate synthetic data with the underlying distributions in the GT data on a variable by variable basis, while accounting for missingness using the “Miss Nodes/States” approach and the latent variable approach.

### Synthetic data compared to ground truth data for underlying distributions

We compare distributions of variables from 100,000 data samples generated by the BN with the original ground truth data under three conditions for handling missing data: first, by simply deleting all cases with missing data. Second, by using “Miss Nodes” (for continuous variables) and “Miss States” (for discrete variables). Finally, by additionally learning latent variables within the BN structure using the FCI algorithm to capture unmeasured effects, including potentially MNAR data.

**Fig. 2:**
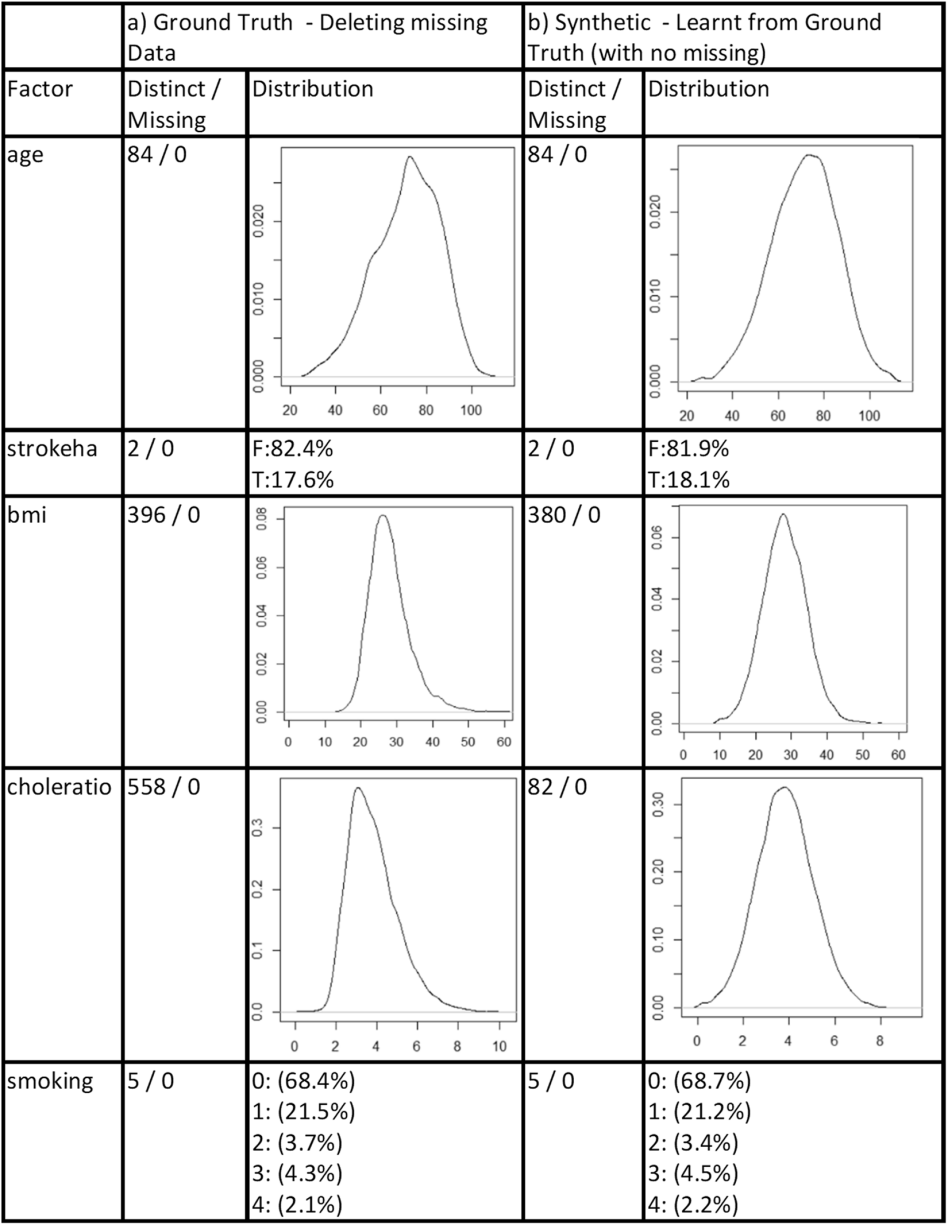
Plots of sample distributions and statistics of the original ground truth data when all missing data are deleted along with plots, distributions, and statistics from the synthetic data that are generated using a BN inferred from the ground truth.

**Fig. 3:**
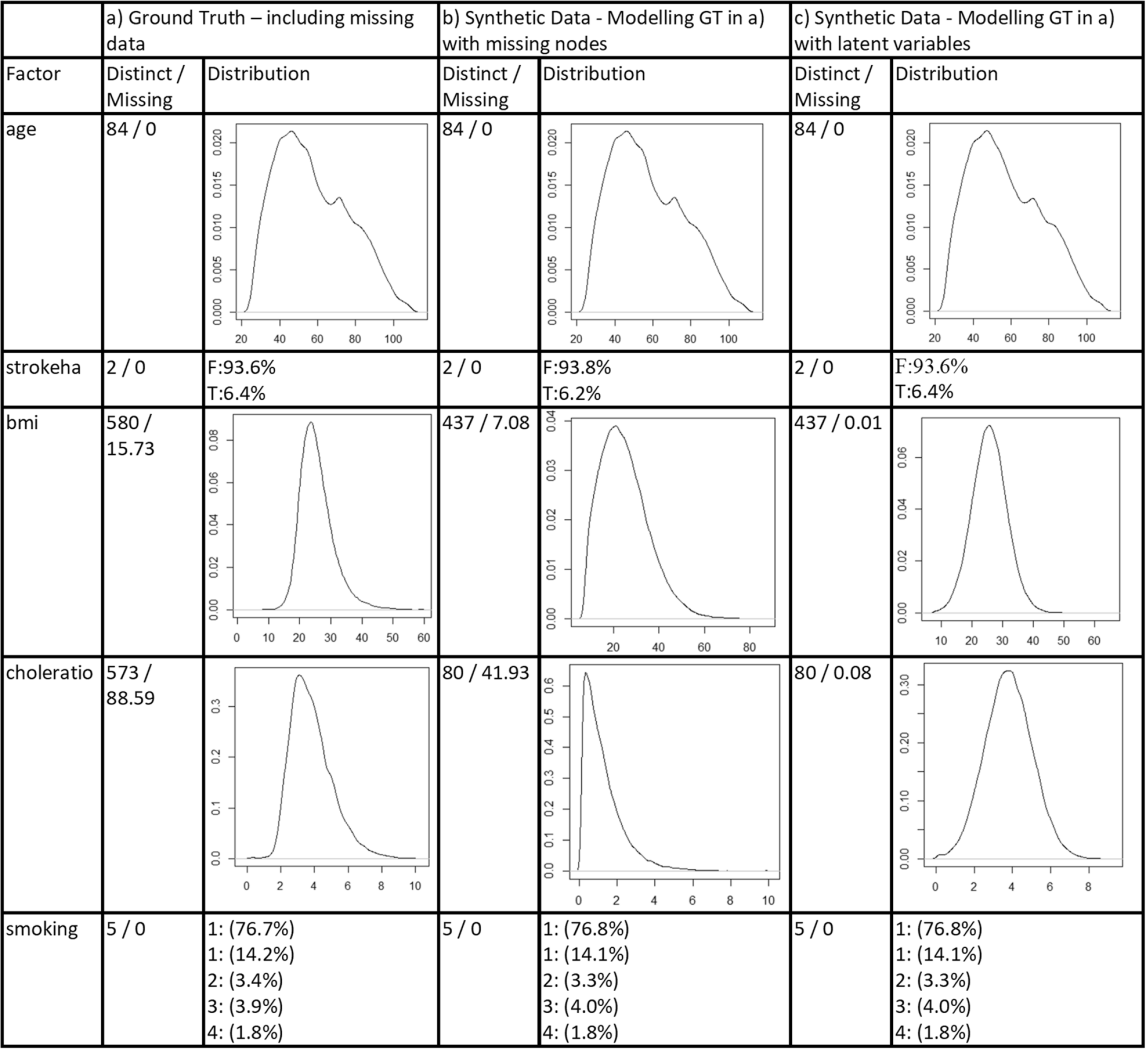
Plots of sample distributions and statistics of the original ground truth data including missing data as well as plots for the synthetic data that models missing data with “Miss Nodes/States” and with latent variables.

Figures 2 and 3 show the resulting distributions for a sample of features in the CPRD. We explore the distribution comparisons between the GT and SYN that is generated by logic sampling from the BN under two conditions for a number of representative variables—first, when missing data are simply deleted (Fig. 2). Figure 2a shows the result for GT and Fig. 2b shows the SYN data generated from this. Second, we explore explicitly modelling the distributions using our approaches described in the “Methods” (Fig. 3). Figure 3a shows the GT with no missing data removed, Fig. 3b shows the SYN data generated from this using our Miss Nodes/States data approach, and Fig. 3c shows the resulting SYN from using the latent variable method. Also included are the number of data points with missing cases and the number of distinct values for a feature (e.g. a value of two for discrete binary features and potentially large numbers for integers and real values).

First, notice how these results show that, for some variables, simply deleting the missing data can result in very different distributions. For example, the age distribution of the GT when missing data are simply removed in Fig. 2a has a very different distribution than for the original GT data without missing data removal in Fig. 3a. What is more, the approach to modelling missingness with “Miss Nodes/States” results in a similar shape distribution to the original in Fig. 3b for some, but in certain cases, the latent variable approach in Fig. 3c results in the most similar distribution to the ground truth with missing data—compare *bmi* in Fig. 3a–c. The bias in categorical data seems less significant and both the “Miss Nodes/States” and latent variable approaches capture the *smoking* and *stroke* distributions very closely though notice how different the distributions are if the missing data are simply removed, highlighting the importance of modelling missing values rather than removing them.

Note that the amount of missing data that is generated (% Missing) is different for the latent variable approach and the “Miss Nodes/States” approach, with the “Miss Nodes/States” approach in Fig. 3b reflecting this value more closely and the latent variable approach exhibiting far fewer missing cases. This is likely due to the latent variable method in Fig. 3c inferring the missing values. In summary, a close distribution can be created between synthetic data sets and ground truth. Distributions are generally closer to the original when missing data are preserved and modelled. We have found this general trend across all features.

Each discrete variable is compared using Chi-squared tests to measure the difference between *n* samples of the *Ground Truth* (GT) and *n* samples of *SYNthetic data* (SYN). For variables with continuous values, Kolmogorov–Smirnov (KS) test is used to measure the distribution difference between GT and SYN data sets. In addition, the Kullback–Leibler divergence (KLD) is used to measure the distribution difference between sampled GT and SYN data sets. These approaches are described in more detail in the “Methods” section.

Chi-squared test is performed with the null hypotheses to (1) test whether there is no significant difference between expected frequencies from SYN and (2) the observed frequencies from GT for each variable (categorical).

Table 1 shows that for all features the null hypothesis cannot be clearly rejected in both scenarios, i.e. removing the missing data and modelling it (all *p* values are far greater than the 0.05 level). This was surprising and implies that simply deleting missing data is not a problem for this primary care data (or at least it does not have much impact on the overall distribution). This could be because of the size of data set that we are dealing with and missing data may be more of an issue with smaller sample sizes. In addition, modelling missingness explicitly is likely to impact certain cases more than others (for example, where people have refused to give certain information for some underlying reason—i.e. MNAR data). These cases may be rare but significant.

**Table 1:** Chi-squared *p* values for the hypothesis of there being a difference in distributions between the GT and SYN data sets for categorical variables.

Variable	Missing deleted *p* value	Missingness modelled (latent) *p* value
strokeha [factor]	0.95	0.36
af [factor]	0.98	0.48
atyantip [factor]	1.00	1.00
steroid [factor]	0.50	0.16
impot [factor]	0.82	0.48
migr [factor]	0.73	0.16
ra [factor]	0.40	0.75
ckidney [factor]	0.90	0.51
semi [factor]	0.65	0.65
sle [factor]	1.00	1.00
treathyp [factor]	0.64	0.28
type1 [factor]	0.51	0.57
type2 [factor]	0.66	0.27
ethr [factor]	0.80	0.92
smoking [factor]	0.84	0.27
fh_cad [factor]	0.57	0.51
gender [factor]	0.87	0.89
region [factor]	0.71	0.28

Chi-squared tests comparing distributions between synthetic and ground truth data for categorical variables.

The KS test is performed to test the hypothesis if the numerical variables of the GT and SYN data sets come from the same distribution. We explore this for a number of different sample sizes (*n*). This is because larger sample sizes make the test more likely to conclude that the two distributions are different (i.e. reject the null hypothesis) because it is very sensitive to differences between distributions^44^.

Table 2 shows that the numerical variables from SYN and GT data sets are indeed from the same distributions (the *p* values are always >0.01, meaning we reject the hypothesis that they are from different distributions) for all variables except *age* and *bmi* for very high sample sizes (indeed the *D* statistics are nearly always smaller for the models using latent variables, which means that data sets are generally closer).

**Table 2:** KS test *p* values for the hypotheses of numerical variables for GT and SYN data sets are from the same distribution and the associated *D* statistic of the test.

Numerical variable	Missingness modelled (latent) *n* = 1000	Missing deleted *n* = 1000	Missingness modelled (latent) *n* = 5000	Missing deleted *n* = 5000	Missingness modelled (latent) *n* = 10,000	Missing deleted *n* = 10,000
*age*	0.023 [*D* = 0.025]	0.302 [*D* = 0.045]	0.017 [*D* = 0.027]	0.261 [*D* = 0.039]	0.008 [*D* = 0.025]	0.017 [*D* = 0.027]
*bmi*	0.023 [*D* = 0.0318]	0.206 [*D* = 0.068]	0.013 [*D* = 0.0315]	0.108 [*D* = 0.071]	0.005 [*D* = 0.0312]	0.014 [*D* = 0.069]
*choleratio*	0.012 [*D* = 0.0793]	0.244 [*D* = 0.046]	0.011 [*D* = 0.0796]	0.138 [*D* = 0.067]	0.009 [*D* = 0.0795]	0.014 [*D* = 0.057]
*sbp*	0.074 [*D* = 0.063]	0.065 [*D* = 0.063]	0.072 [*D* = 0.063]	0.064 [*D* = 0.063]	0.071 [*D* = 0.063]	0.062 [*D* = 0.063]
*sbps*	0.082 [*D* = 0.0340]	0.081 [*D* = 0.083]	0.080 [*D* = 0.0358]	0.072 [*D* = 0.076]	0.042 [*D* = 0.0348]	0.028 [*D* = 0.073]

Population sizes are 1,000, 5,000, and 10,000. Kolmogorov–Smirnoff tests comparing distributions between synthetic and ground truth data for numerical variables.

We now look at using the KLD to see the difference over all variables for different samples (of size 100,000) of GT data in comparison to the difference between SYN and GT data sets.

Table 3 shows the mean squared KL distances between repeated GT samples compared to SYN samples scored against GT samples. Table 4 calculates the ***diff***_**KL**_ values using the results above. Additionally, the missing data rates of continuous variables are listed below based on the KL distance. Applying a KS test to these results for each variable shows that the KL distances of two ground truth samples is not significantly different to the KL distance between a ground truth sample and a synthetic data samples for variables with reasonably higher distances (*chol* and *bmi* with *p* values of 0.168 and 0.052, respectively). For *age* (*p* = 0.0), *sbp* (*p* = 0.0), and *sbps* (*p* = 0.002), they were found to be significantly different*: age* and *sbps* distances are very small (or zero) for both GT and SYN data comparisons (see Table 3) and *sbp* interestingly is the variable where the synthetic data actually contains smaller distances to ground truth than between ground truth samples.

**Table 3:** The mean squared ${\boldsymbol{D}}_{{\boldsymbol{KL}}}({\boldsymbol{GT}}_{\boldsymbol{i}}||{\boldsymbol{GT}}_{\boldsymbol{i}}^{\boldsymbol{n}})$ (*n* ∈ {1…10}) of each variable ending with “gt” and the mean squared ${\boldsymbol{D}}_{{\boldsymbol{KL}}}({\boldsymbol{GT}}_{\boldsymbol{i}}^{\boldsymbol{m}})\left. {\left\| {{\boldsymbol{SY}}_{\boldsymbol{i}}^{\boldsymbol{n}}} \right.} \right)$ (*m*,*n* ∈ {1…10}) of each variable ending with “sy”.

Iteration	age_gt	chol_gt	bmi_gt	sbp_gt	sbps_gt	age_sy	chol_sy	bmi_sy	sbp_sy	sbps_sy
1	0.000	46.662	4.995	5.395	0.652	0.002	56.599	9.007	4.550	1.576
2	0.000	48.260	3.403	5.520	0.634	0.002	88.224	3.702	5.747	1.521
3	0.000	57.847	2.407	6.203	0.623	0.002	46.680	11.784	4.420	1.580
4	0.000	47.924	2.657	5.194	0.644	0.002	36.679	10.985	4.735	0.998
5	0.000	51.721	9.957	5.497	0.639	0.002	56.610	2.923	3.814	1.500
6	0.000	51.067	3.254	5.888	0.734	0.002	46.622	11.882	3.898	1.288
7	0.000	44.377	3.373	6.007	0.680	0.002	68.853	6.966	3.961	1.458
8	0.000	46.583	4.790	5.351	0.731	0.002	70.475	11.548	5.645	1.369
9	0.000	53.491	2.784	6.664	0.614	0.002	62.019	2.900	3.645	1.572
10	0.000	51.239	2.896	5.999	0.642	0.002	48.852	12.751	4.445	1.261

The mean squared Kullback–Leibler divergence between resampled ground truth data compared to synthetic samples scored against ground truth.

**Table 4:** KL divergence differences between resampled data sets and synthetic data sets for each variable and associated missing rate in parentheses.

Iteration	*diff*_*KL*_*age* (0% missing)	*diff*_*KL*_*chol* (88.47%)	*diff*_*KL*_*bmi* (15.85%)	*diff*_*KL*_*sbp* (8.37%)	*diff*_*KL*_*sbps* (38.25%)
1	0.002	9.937	4.012	−0.845	0.924
2	0.002	39.964	0.299	0.227	0.887
3	0.002	−11.167	9.377	−1.783	0.957
4	0.002	−11.245	8.328	−0.459	0.354
5	0.002	4.889	−7.034	−1.683	0.861
6	0.002	−4.445	8.628	−1.99	0.554
7	0.002	24.476	3.593	−2.046	0.778
8	0.002	23.892	6.758	0.294	0.638
9	0.002	8.528	0.116	−3.019	0.958
10	0.002	−2.387	9.855	−1.554	0.619
Mean (SD)	0.002 (0.000)	8.244 (16.863)	4.393 (5.376)	−1.286 (1.065)	0.753 (0.204)

Kullback–Leibler divergence differences between resampled ground truth and synthetic data.

We assume that that the synthetic data are suitably similar in distribution to the ground truth if the KL distances of the samples of synthetic data to the ground truth are similar to the KL distances of the resamples of ground truth data between one another. In order to test this, we randomly resample from ground truth, GT, and calculate the KL distances between each sample. These distances are then compared to the KL distances between the synthetic data, SYN, and the GT. ***diff***_**KL**_ represents the difference in the KL distances between multiple resamples of GT and between SYN data and GT, for each variable.

The mean ***diff***_**KL**_ values for the tested variables (in bottom rows of Table 4) indicate that the synthetic KLDs vary between 8.244 and −1.286 when missing data are presented. In some cases, such as systolic blood pressure (*sbp*), the synthetic data are constantly closer to the ground truth distribution shape than the resampled data are to one another. For variables without missing values such as age, the KL distance differences are close to zero. In other words, the synthetic data are closer to the ***GT***_***i***_ distributions. We can thus conclude that our approach generates synthetic data that is no more different to the ground truth data than differences found when generating multiple resamples of ground truth.

We now explore the joint distributions in the synthetic data sets by using kernel maximum mean discrepancy (kMMD) with a radial basis function kernel. We conducted a combination of distribution tests for 2-variable (253 combinations), 3-variable (1771 combinations), and 4-variable (8855 combinations) comparison. The hypothesis *H*_0_ for kMMD is that samples to be tested come from the same distribution with alpha ~0.05. With the same SYN data sets from the previous experiment for each iteration (10 iterations), we aim to see the difference between same-sized samples from the GT population and samples from SYN in terms of their distributions. The results of the *H*_0_ acceptance rate are shown in Table 5 (joint distribution tests on 1000 samples from 1 million GT population and 100,000 sampled SYN data).

**Table 5:** Joint distribution tests for 2-, 3-, and 4-variable combinations using kernel MMD.

Iteration	2-kMMD	3-kMMD	4-kMMD
1	75.49%	65.50%	56.82%
2	76.68%	68.44%	61.69%
3	69.96%	61.10%	53.64%
4	75.89%	66.57%	58.78%
5	83.00%	75.21%	68.02%
6	75.89%	67.25%	59.35%
7	75.89%	67.08%	58.80%
8	75.89%	67.65%	58.92%
9	69.96%	60.42%	53.19%
10	75.49%	66.12%	57.09%

Joint distribution similarity for synthetic and ground truth data. In each iteration, 1000 data instances are sampled from ground truth population of 1 million instances and another 1000 from synthetic data set. The results of *H*_0_ being not rejected are shown in percentages, and average *H*_0_ acceptance rates are 75.42, 66.53, and 58.63%, respectively.

We can conclude from these results that the distance between SYN and GT distributions are generally low when taking account of low-dimensional combinations of data features. What is more, they are not significantly worse than between two GT samples, when using our proposed methods of latent variable modelling to handle missingness. The distance between SYN and GT, however, can increase as the number of combinations of data features increases (potentially as a result of simplification within the structure of the model).

In order to see the practical implication of differences between GT and SYN data, we further compare GT and SYN’s performance on training and testing machine learning classifiers in the next section.

### Synthetic data compared to ground truth data for machine learning classifier comparison

**Fig. 4:**
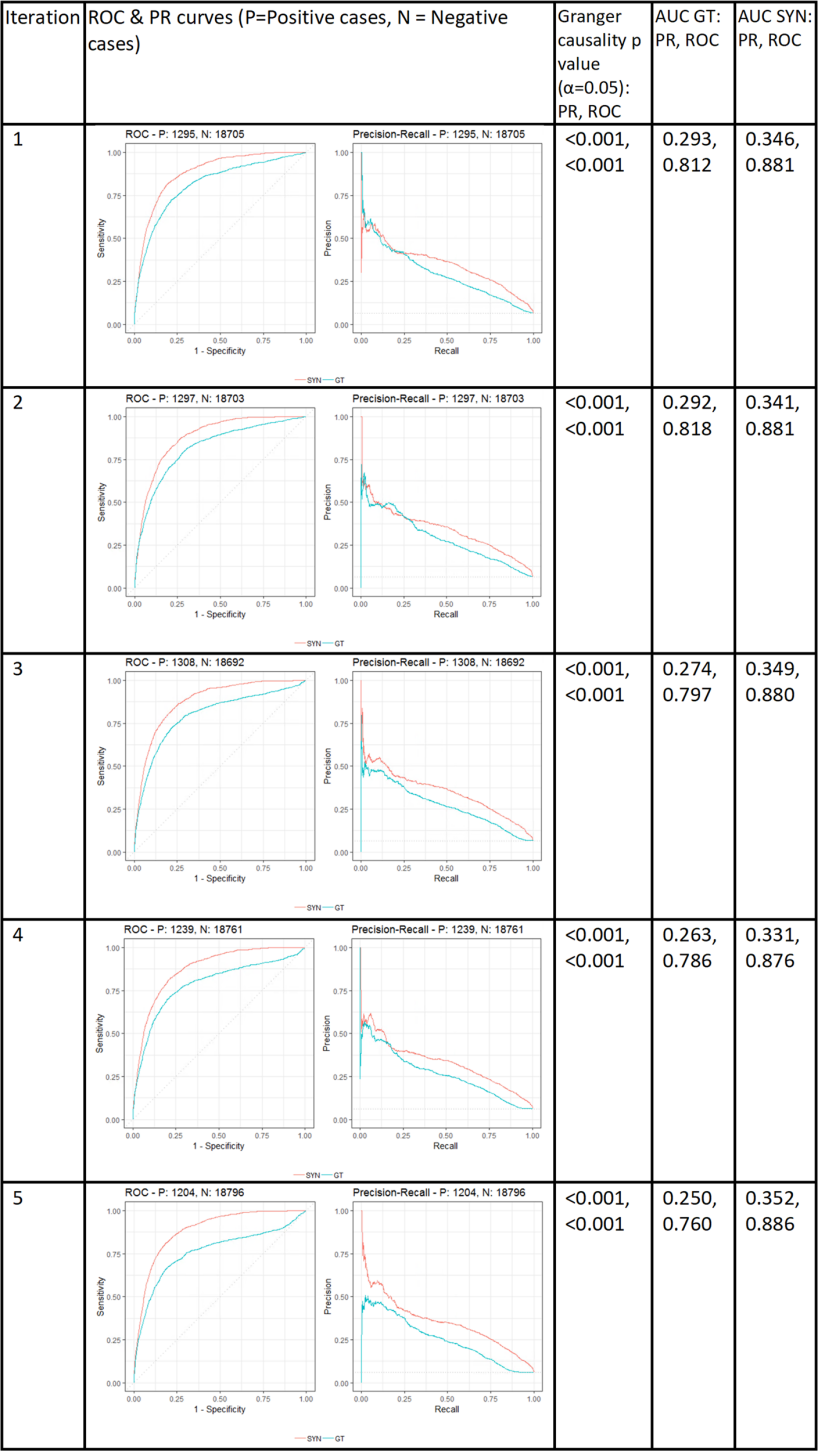
Five-sample sensitivity analyses for a Bayesian generalised linear classifier on GT and SYN data (latent model) for fixed sample size of 100,000, including ROC and PR curves, and AUC and Granger statistics.

Figure 4 compares the receiver operator characteristic (ROC) and precision recall (PR) curves for the GT data and SYN data (generated using the latent variable method) when a machine learning classifier is inferred for predicting stroke. The results shown are on a Bayesian generalised linear classifier. In particular, the area under the ROC curve (AUC) for both curves is calculated for GT and SYN samples and the Granger causality statistic as described in the “Methods” is calculated to determine how predictive the SYN curves are of the underlying GT curves. Note that a *p* value is generated that determines the Granger causality statistic at the 5% significance level.

First, notice that the ROC and PR curves are similar in shape for the GT data (blue) and the SYN data (red). Observing these sample curves, it is not surprising the Granger causality statistic for all samples is significant at less than the *p* = 0.001 level. We also applied identical tests to other machine learning classifiers (see Supplementary Figs. 3 and 4) where all *p* values were found to be <0.001 except for ROC curves generated when using stepwise regression with *N* < 1000. We conclude that the outcome of using SYN data samples for the selected prediction algorithms is that we can predict the sensitivity analysis of using actual GT data (as their difference is not significant). Indeed, this experiment set-up implies that the generated SYN data are able to achieve equivalent statistical results to GT data. (Incidentally, these AUC results are in line with similar results documented by Ozenne et al.^45^ i.e. high AUC ROC and low AUC PR curves were observed across tests.).

### Detecting re-identification risks using outlier analysis with distance metrics

Finally, we explore the risk of re-identification of patients from the SY data based on the clones (*R*_clone_), inliers (*N*_in_), and outlier (*N*_out_) statistics described in the “Methods” section. We base our experiments on the concept of event per variable (EPV), which explores the effect of sample size and number of variables on predictive accuracy^46^. The number of EPV is the number of events divided by the number of degrees of freedom required to represent all of the variables in the model. We use an EPV value of 22.2 based on the conclusions in the study by Austin and Steyerberg^46^. The results in Table 6 below are based on 10 iterations of resampling without replacement. This indicates a sample size of 7000 for each iteration within 11 random population groups. Notice how the risk of clones decreases as the sample size increases (as one would expect). While we also see that the risk of outliers decreases, they are always very small. What is more, the actual number of outliers generated stays relatively stable (between 10 and 70). These statistics demonstrate that, while there is always a risk of risk of a synthetic patient being linked to an actual patient in the ground truth data in the case or outliers, we can exploit such metrics to identify the at-risk samples and make a decision as to whether they should be removed or not (if they are clones or outliers with a too-small *k*-anonymisation value).

**Table 6:** The risk of seeing clones *R*_clone_, inliers *N*_in_, and outliers *N*_out_ in the synthetic data for increasing samples sizes of ground truth data.

GT population size	*R*_clone_	*R*_in_, Pr = 0.001	*R*_out_, Pt = 0.999
100,000	0.016	462 (0.4620%)	25 (0.0250%)
200,000	0.013	770 (0.3850%)	34 (0.0170%)
300,000	0.014	613 (0.2043%)	24 (0.0080%)
400,000	0.012	553 (0.1383%)	53 (0.0133%)
500,000	0.016	529 (0.1058%)	19 (0.0038%)
600,000	0.009	254 (0.0423%)	45 (0.0075%)
700,000	0.008	534 (0.0763%)	24 (0.0034%)
800,000	0.011	581 (0.0726%)	13 (0.0016%)
900,000	0.012	518 (0.0576%)	33 (0.0037%)
1,000,000	0.012	30 (0.0030%)	45 (0.0045%)
2,000,000	0.010	78 (0.0039%)	29 (0.0015%)

Risk of seeing clones, inliers, and outliers.

## Discussion

This paper has introduced and validated a set of techniques to model complex heterogeneous data for generating realistic synthetic data sets that capture the correct dependencies and distributions. The approach exploits resampling with probabilistic graphical modelling that explicitly handles missingness and complex non-linear/non-Gaussian relationships and is transparent in how data are modelled enabling biases to be assessed and accounted for. Through a case study on cardiovascular risk, the paper has demonstrated that these synthetic data sets not only generate similar distributions over both discrete and continuous variables but also produce similar sensitivity analyses to the original ground truth data (in the form of PR and ROC curves).

Patient privacy is quantified through a demonstration that the proximity of individual synthetic data points to real patients can be scored by using outlier statistics and distance metrics, though more research is required on the robustness of this particularly when clusters of patients with rare disease/demographics are modelled. We have demonstrated that our method can flag identical or similar patient profiles in the synthetic and real data. While the occurrence of these “clones” or similar rare patient profiles appears to be low (and does not seem to increase with sample size), there is still a small risk. However, our metrics enable these risks to be quantified so that appropriate action can be taken prior to releasing any data (depending on the risk protocol adopted).

**Fig. 5:**
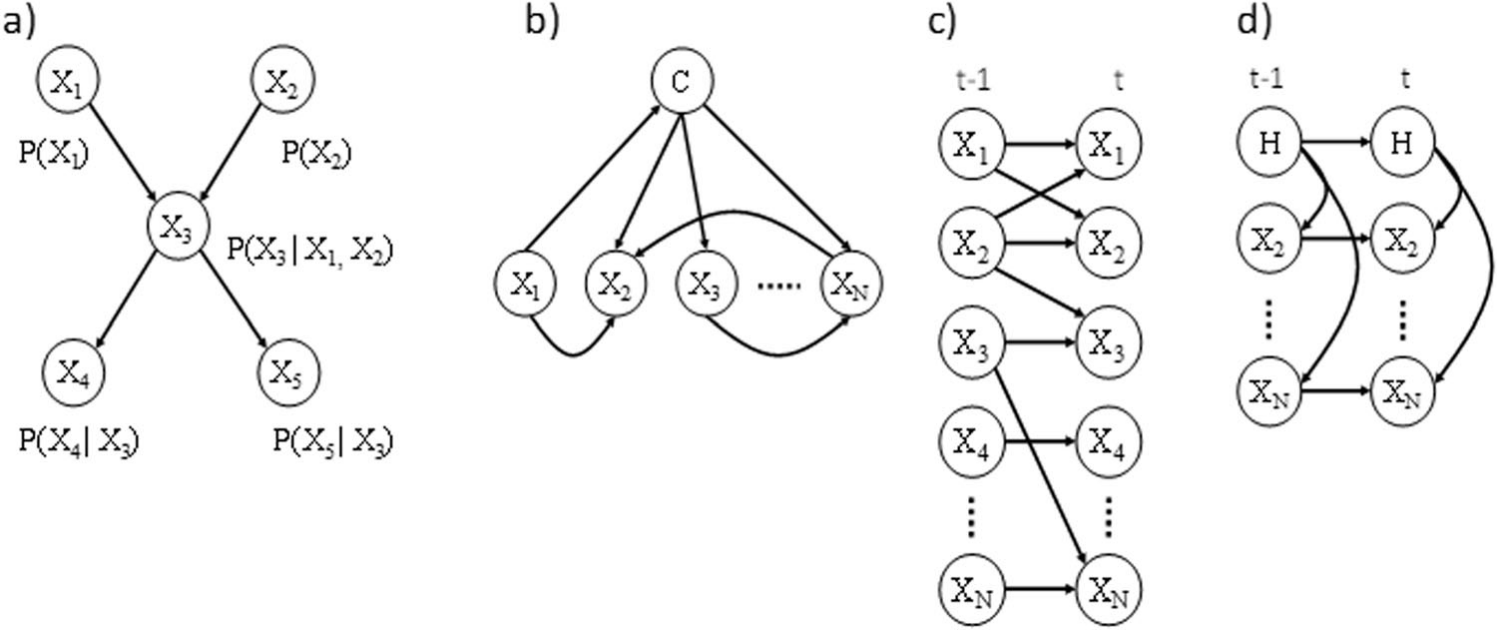
Bayesian network architectures. **a** A Bayesian network with four nodes. **b** A Bayesian network classifier with class node *C.* **c** A dynamic Bayesian network with two time-slices, *t* and *t*−1. **d** A Hidden Markov model with latent variable *H*.

Another issue that may impact the production of realistic synthetic data is the temporal nature of many health data sets. The methods that we have adopted here are well suited to handle this characteristic. For example, the dynamic BN^47^ and hidden Markov model^48^ are generalisations of the standard BN model used in this paper. Here the time dimension is represented by unrolling networks so that nodes represent variables at specific time points in Fig. 5c, d. These approaches will be included in our future directions for the project.

Generating synthetic data from large-scale real-world data that are noisy, contain structurally missing data, and many non-linear relationships such as the UK primary care data can bring enormous benefits to AI research. In particular, it can prevent the need for using real patient data when developing and validating state-of-the-art predictive models. This paper has explored several key issues involved with this but there is scope for more research to ensure that these data sets do not contain underlying biases (e.g. by exploring data collection processes) or present a privacy risk (e.g. by carrying out simulated privacy attacks), if they are to be made freely available without any access controls to facilitate innovation.

## Methods

### Data description—CPRD Aurum

For our case study, we used an extract from this database on 122,328 patients (all aged >16 years).

We tested the synthetic data performance using a risk prediction algorithm for cardiovascular disease (encompassing stroke, transient ischaemic attack, myocardial infarction, heart attacks, and angina). We used the same features as used by Hippisley-Cox et al.^49^ for predicting the onset of cardiovascular disease within 10 years (explained in Table 7).

**Table 7:** Description of the selected features used from CPRD for analysis based on predicting cardiovascular disease.

Variable acronym	Type of variable (D = dependent, I = independent)	Description
*age*	I	Age of patient
*gender*	I	Gender of patient
*strokeha*	D	Stroke or heart attack
*af*	I	Atrial fibrillation
*atyantip*	I	On atypical antipsychotic medication?
*steroid*	I	On regular steroid tablets?
*impot*	I	A diagnosis of or treatment for erectile dysfunction?
*migr*	I	Do you have migraines?
*ra*	I	Rheumatoid arthritis?
*ckidney*	I	Chronic kidney disease (stage 3, 4, or 5)?
*semi*	I	Severe mental illness? (this includes schizophrenia, bipolar disorder, and moderate/severe depression)
*sle*	I	Systemic lupus erythematosus?
*treathyp*	I	On blood pressure treatment?
*type1*	I	Type I diabetes
*type2*	I	Type II diabetes
*bmi*	I	Body mass index
*ethr*	I	Ethnicity
*choleratio*	I	Cholesterol/HDL ratio
*sbp*	I	Systolic blood pressure (mmHg)
*sbps*	I	Standard deviation of at least two most recent systolic blood pressure readings
*smoking*	I	Smoking status
*fh_cad*	I	Family history of coronary artery disease
*region*	I	Practice region

Selected features for predicting cardiovascular disease from the CPRD.

### BN modelling

We have selected a BN due to its flexibility and transparency—see Fig. 5a. BNs model the joint distribution of a data set *p(X)* by making assumptions about conditional independence between features that are captured in a directed acyclic graph (DAG). A BN represents the joint probability distribution over a set of variables, *X*_1_,…,*X*_*N*_, by exploiting conditional independence relationships. These relationships are represented by a DAG. The conditional probability distribution (CPD) associated with each variable, *X*_*i*_, encodes the probability of observing its values given the values of its parents and can be described by a continuous or a discrete distribution. All the CPDs in a BN together provide an efficient factorisation of the joint probability (see Eq. 1)

1 $$p\left( x \right) = \mathop {\prod }\limits_{i = 1}^n p(x_i|{\rm{pa}}_i),$$

where pa_*i*_ are the parents of the node *x*_*i*_ (which denotes both node and variable).

This family of models can be used to perform inference by entering evidence into one or more nodes and inferring the posterior distributions of the remaining nodes. In this way, data can be sampled under different observations. We use logic sampling^50^ to sample data where we “fix” certain features if necessary, by entering evidence. For example, we can generate data where all samples are formed from people aged >65 years, or female-only samples, or all people who have been diagnosed with hypertension.

BNs can be constructed by hand where the links represent some form of influence or they can be inferred from data using constraint-based algorithms such as the PC or FCI algorithm^20^, or search and score methods such as BIC^51^, or MDL^52^. Here we use a method to infer models directly from the CPRD that can handle missing data known as structural expectation maximisation^26,53^. We record the fit of the models over multiple runs to calibrate the robustness of the models to sampling variation. This family of models can be used to perform machine learning prediction such as in the BN classifier in Fig. 5b, clustering using the EM algorithm, and time-series forecasting by unrolling the BN into the time-domain in Fig. 5c, d.

**Fig. 6:**
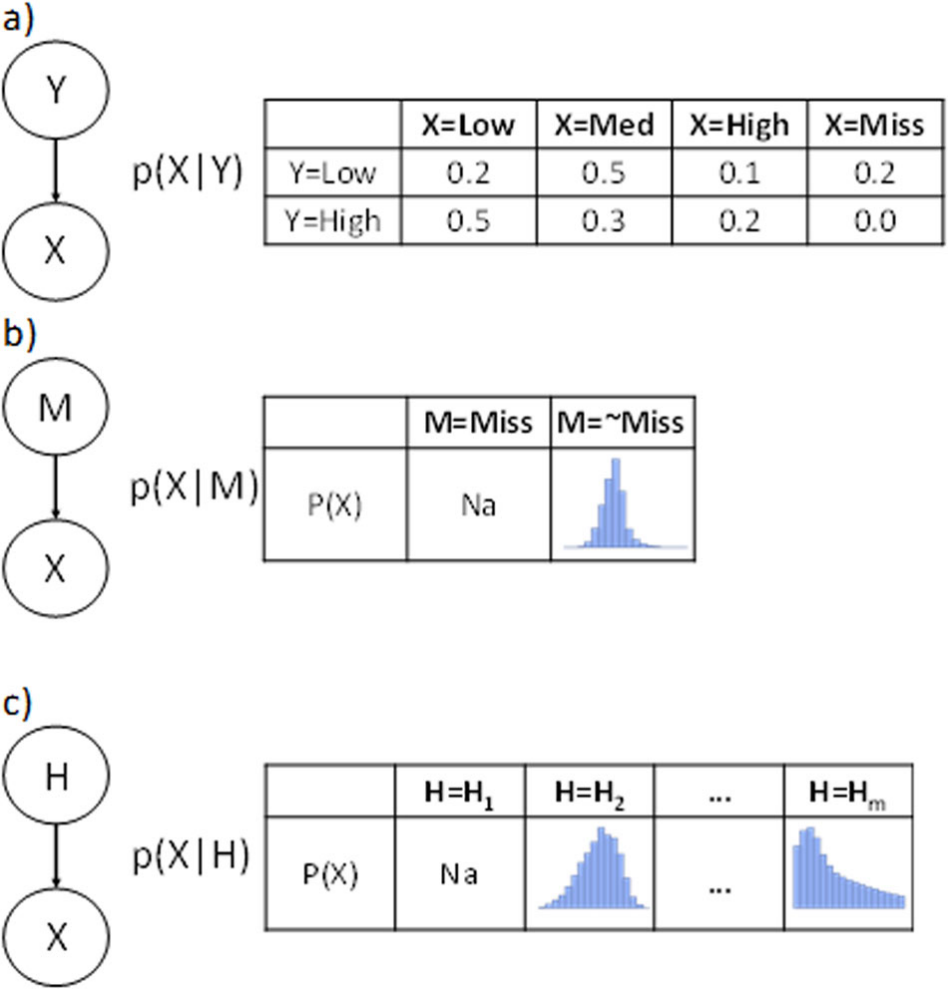
Methods to capture missing data and unmeasured effects. **a** A binary “Miss Node” pointing to all continuous nodes in a Bayesian network. **b** A “Miss State” for discrete nodes. **c** A latent variable with *m* states to capture Missing Not at Random data and other unmeasured effects (in both discrete and continuous nodes).

We use three approaches to handle missing data: one for discrete nodes where we add a “missing” state to all possible states in Fig. 6a, one for continuous nodes where we add a new binary parent to each node that represents either missing or not in Fig. 6b and one where we use the FCI algorithm to infer any latent variables in the network. The algorithm is applied to 10 resampled data sets to calculate robust statistics for determining the inclusion and position of any latent variables in the networks, e.g. Fig. 6c where the distribution of a variable is directly influenced by a discrete latent variable that is discovered as a parent. By identifying these robust latent variables, we aim to improve the details of the underlying distributions as well as capture any MNAR effects. Please see Supplementary Fig. 1 for the threshold statistics for each variable and Supplementary Fig. 2 for a sample network including latent variables.

### Experiments

#### Modelling missing data to capture underlying distributions

We assume that the synthetic data are suitably similar in distribution to the ground truth if the KL distances of the samples of synthetic data to the ground truth are similar to the KL distances of the resamples of ground truth data between one another. In order to test this, the experiment base population ***GT***_*i*_ is randomly sampled from the full CPRD primary care database. KL distances are compared to assess if the generated SYN can be representative. Three groups of data are used, where *i* denotes the sample size.

***GT***_***i***_—The sampled ground truth from the total population *P*;

${\boldsymbol{SY}}_{\boldsymbol{i}}^{\boldsymbol{n}}$—The generated *n* synthetic data sets based on ***GT***_***i***_ (with equal size to ***GT***_***i***_);

${\boldsymbol{GT}}_{\boldsymbol{i}}^{\boldsymbol{n}},{\boldsymbol{GT}}_{\boldsymbol{i}}^{\boldsymbol{m}}$—The other *n* or *m* sets of resampled ground truth data (with equal size to ***GT***_***i***_) from the total population *P* without replacement.

Two KL distances are obtained from each target variable’s distribution shape and these then can be compared as in Eqs. 2–5.

2 $$\overline {{\boldsymbol{D}}_{{\boldsymbol{KL}}}^2} ({\boldsymbol{GT}}_{\boldsymbol{i}}^{\boldsymbol{m}}||{\boldsymbol{SY}}_{\boldsymbol{i}}^{\boldsymbol{n}})\;{\mathrm{and}}\;\overline {{\boldsymbol{D}}_{{\boldsymbol{KL}}}^2} ({\boldsymbol{GT}}_{\boldsymbol{i}}||{\boldsymbol{GT}}_{\boldsymbol{i}}^{\boldsymbol{n}}).$$

- When the $\overline {{\boldsymbol{D}}_{{\boldsymbol{KL}}}^2}$is close to 0, then the distributions are almost identical.
- When the value of $\overline {{\boldsymbol{D}}_{{\boldsymbol{KL}}}^2} ({\boldsymbol{GT}}_{\boldsymbol{i}}^{\boldsymbol{m}}||{\boldsymbol{SY}}_{\boldsymbol{i}}^{\boldsymbol{n}})$ is close to $\overline {{\boldsymbol{D}}_{{\boldsymbol{KL}}}^2} ({\boldsymbol{GT}}_{\boldsymbol{i}}||{\boldsymbol{GT}}_{\boldsymbol{i}}^{\boldsymbol{n}})$, then the generated synthetic variable has an almost identical distribution as the ***GT***_***i***_.

3 $$\overline {{\boldsymbol{D}}_{{\boldsymbol{KL}}}^2} ({\boldsymbol{GT}}_{\boldsymbol{i}}||{\boldsymbol{GT}}_{\boldsymbol{i}}^{\boldsymbol{n}})\;{\mathrm{is}}\;{\mathrm{the}}\;{\mathrm{mean}}\;{\mathrm{squared}}\;{\mathrm{KLD}}\;\left( {n \in \left\{ {1 \ldots 10} \right\}} \right).$$

4 $$\overline {{\boldsymbol{D}}_{{\boldsymbol{KL}}}^2} ({\boldsymbol{GT}}_{\boldsymbol{i}}^{\boldsymbol{m}}||{\boldsymbol{SY}}_{\boldsymbol{i}}^{\boldsymbol{n}})\;{\mathrm{is}}\;{\mathrm{the}}\;{\mathrm{mean}}\;{\mathrm{squared}}\;{\mathrm{KLD}}\;\left( {m,n \in \left\{ {1 \ldots 10} \right\}} \right).$$

5 $${\boldsymbol{diff}}_{{\boldsymbol{KL}}} = \overline {{\boldsymbol{D}}_{{\boldsymbol{KL}}}^2} ({\boldsymbol{GT}}_{\boldsymbol{i}}^{\boldsymbol{m}}||{\boldsymbol{SY}}_{\boldsymbol{i}}^{\boldsymbol{n}}) - \overline {{\boldsymbol{D}}_{{\boldsymbol{KL}}}^2} ({\boldsymbol{GT}}_{\boldsymbol{i}}||{\boldsymbol{GT}}_{\boldsymbol{i}}^{\boldsymbol{n}}).$$

We also explore the joint distribution of our models compared to the ground truth data using MMD. The MMD is an approach to represent distances between distributions as distances between mean embeddings of features^54^. The approach tests whether distributions *p* and *q* are different on the basis of samples drawn from each of them, by finding a smooth function that is large on the points drawn from *p* and small (as negative as possible) on the points from *q*. The test statistic is the difference between the mean function values on the two samples. When this is large, the samples are likely to be drawn from different distributions.

For example, if we have any joint distributions *P* from ***GT***_***i***_ and *Q* from ${\boldsymbol{SY}}_{\boldsymbol{i}}^{\boldsymbol{n}}$ over a set ***X***. The MMD can be defined by a feature map φ:*X*→*H*, where *H* is called a reproducing kernel Hilbert space. Hence, when $x = H = R_{\rm{d}}\;{\mathrm{and}}\;\varphi \left( x \right) = {\rm{a}}\;{\rm{kernel}}\;{\rm{function}}\;{\rm{over}}\;x$. MMD is defined in Eq. 6.

6 $$\begin{array}{l}{\mathrm{MMD}}\left( {P,Q} \right) = ||E_{X \sim P}\left[ {\varphi \left( X \right)} \right] - E_{Y \sim Q}\left[ {\varphi \left( Y \right)} \right]||_H\\ \qquad\qquad\quad\;\;\;= ||E_{X \sim P}[X] - E_{Y \sim Q}[Y]||R_{\rm{d}}\\\qquad\qquad\quad\;\;\; = ||\mu P - \mu Q||R_{\rm{d}},\end{array}$$

where *μP* and *μQ* are the mean embeddings for distributions *p* and *q*.

We take 10 synthetic and ground truth data set pairs. For each pair, we explored the combination of 2, 3, and 4 variables and applied the MMD test to compare all combinations of these variables. Each test produces the *H*_0_ hypothesis for that combination. We calculate the percentage of times that the *H*_0_ is not rejected for the combinations of 2, 3, and 4 variables.

#### Comparing machine learning classifiers inferred and tested from synthetic data and ground truth data

ROC and PR curves are often used to assess the predictive performance of a machine learning model. ROC curves capture the trade-off between false positives and false negatives but can often mask the biases in imbalanced data sets (for example, when the positive case is rare in a population)^55^. PR curves, on the other hand, can detect these biases as they capture the trade-off between precision (also known as the positive predictive value representing the number of correct true positives from all positive prediction) and recall (sensitivity). We analyse the ROC curves and PR curves that are generated when 3 machine learning classifiers (stepwise regression, linear discriminant analysis, and Bayesian generalised linear models) are used to model and predict GT data. We explore the ROC and PR plots for the classifiers’ performance on the SYN data and the original GT. We also measure the capability of the synthetic data curves to predict the GT curves for varying sample sizes using a Granger causality test^56^. In our experiments, the Granger causality test checks for the null hypothesis that the synthetic data curves cannot predict (or “Granger cause”) the ground truth curves.

#### Detecting re-identification risks using outlier analysis with distance metrics

The method we propose aims to generate synthetic data that avoids privacy issues associated with releasing real patient data. However, if the synthetic data sets enable re-identification of real patients (for example, through proximity between a synthetic data point and a real patient), then the intrinsic value is lost. As the probability of re-identification increases, the more unique a patient’s data is (for example, the older a patient is or cases of rare disease). Here we use a form of outlier detection to measure this risk. We randomly select synthetic datapoints from SYN and calculate the distances between it and all GT datapoints. Using an outlier analysis method (based on the distribution of GT data and the individual synthetic data), we calculate the number of GT datapoints (*k*) that are in the same distribution as the synthetic data point (rather than being statistically separate as an outlier). We apply this for varying large samples (100 K to 1 million) of synthetic datapoints. The smallest value of *k* for each of these can be considered the *k*-anonymisation value.

We use the quantile function to assess how many real-world patients are close to a synthetic patient given a pre-defined probability of smallest distance (e.g. Euclidean distance) observations. For example, given the probability of 0.1%, *n* observations of real patient records that are closest to a real patient record can be obtained. In this experiment, GT and SYN data sets are combined into one data set, so the total size of the data set will be *S* = *S*_GT_ = *S*_SYN_, and we define the instances with high privacy risk under any of the following conditions:

Clones—when distance is 0, i.e. the synthetic patient record is identical to real-world patient record, the clone rate is used to measure clone risk *R*_clone_ defined in Eq. 7.

7 $$R_{{\rm{clone}}} = \frac{{{\rm{Total}}\;{\rm{identical}}\;{\rm{instances}}}}{{{\rm{Total}}\;{\rm{instances}}}}.$$

Inliers—when there is only one real patient instance that is closest to the synthetic patient given a pre-defined probability Pr within lower quantiles. The total number of such pairs are used to measure inliers risk *R*_in_ defined in Eq. 8.

8 $$R_{{\rm{in}}} = |\left( {{\rm{Pair}}\left( {{\rm{SYN}}_i,{\rm{GT}}_j} \right)|{\rm{Pr}}} \right)|,i,j \in \{ 1, \ldots ,S\}.$$

Outliers—when there is only one real patient instance that is closest to the synthetic patient given a pre-defined probability Pt within upper quantiles. The total number of such pairs are used to measure outliers risk *R*_out_ defined in Eq. 9.

9 $$R_{{\rm{out}}} = |\left( {{\rm{Pair}}\left( {{\rm{SYN}}_i,{\rm{GT}}_j} \right)|{\rm{Pt}}} \right)|,i,j \in \{ 1, \ldots ,S\}.$$

#### Ethics

The project was undertaken within the institutional governance framework of the Medicines and Healthcare products Regulatory Agency (MHRA) UK and Brunel University London. The use of real anonymised patient data as ground truth data was undertaken under the CPRD’s overarching research ethics committee (REC) approval (reference: 05/MRE04/87) and within CPRD’s secure research environment. Additional advice on privacy of the ground truth data was obtained from the UK Information Commissioner’s Office (ICO) Innovation Hub in response to a formal query by the MHRA.

### Reporting summary

Further information on research design is available in the Nature Research Reporting Summary linked to this article.

## Supplementary information

Supplementary Information

Reporting Summary Checklist

## Data Availability

Access to anonymised patient data from CPRD is subject to a data sharing agreement (DSA) containing detailed terms and conditions of use following protocol approval from CPRD’s Independent Scientific Advisory Committee (ISAC). The generated synthetic data set discussed in this paper can also be requested from CPRD subject to a DSA (https://www.cprd.com/content/synthetic-data).

## Code availability

All our *R* code is available via Github (https://github.com/zhenchenwang/latent_model). The *R* package *bnlearn* (v4.6.1) is used for all Bayesian network inference. The R function *FCI* is used, which is part of the *pcalg* package (v2.6–11), to identify latent variables. *Kmmd* is implemented using the *R* Package *kernlab* (v0.9–29).
