# Supplementary material for: Generating high-fidelity synthetic patient data for assessing machine learning healthcare software
Source: NPJ Digit Med. 2020 Nov 9;3:147. doi: 10.1038/s41746-020-00353-9 (PMC7653933; doi:10.1038/s41746-020-00353-9)
Supplement: Supplementary file 1 — Supplementary Information [file 41746_2020_353_MOESM1_ESM.pdf]

Supplementary Figure 1. The Confidence assigned to links between factors in the Ground Truth and discovered latent variables calculated using bootstrap. The confidence for each variable (green bars) represents the number of times that a latent variable has been discovered as a parent in conjunction with other Ground Truth data variables. Those latent variables and links above a specified threshold are included in the final model. The threshold is set at 0.9 for continuous variables and 0.7 for discrete (shown as a red dotted line).

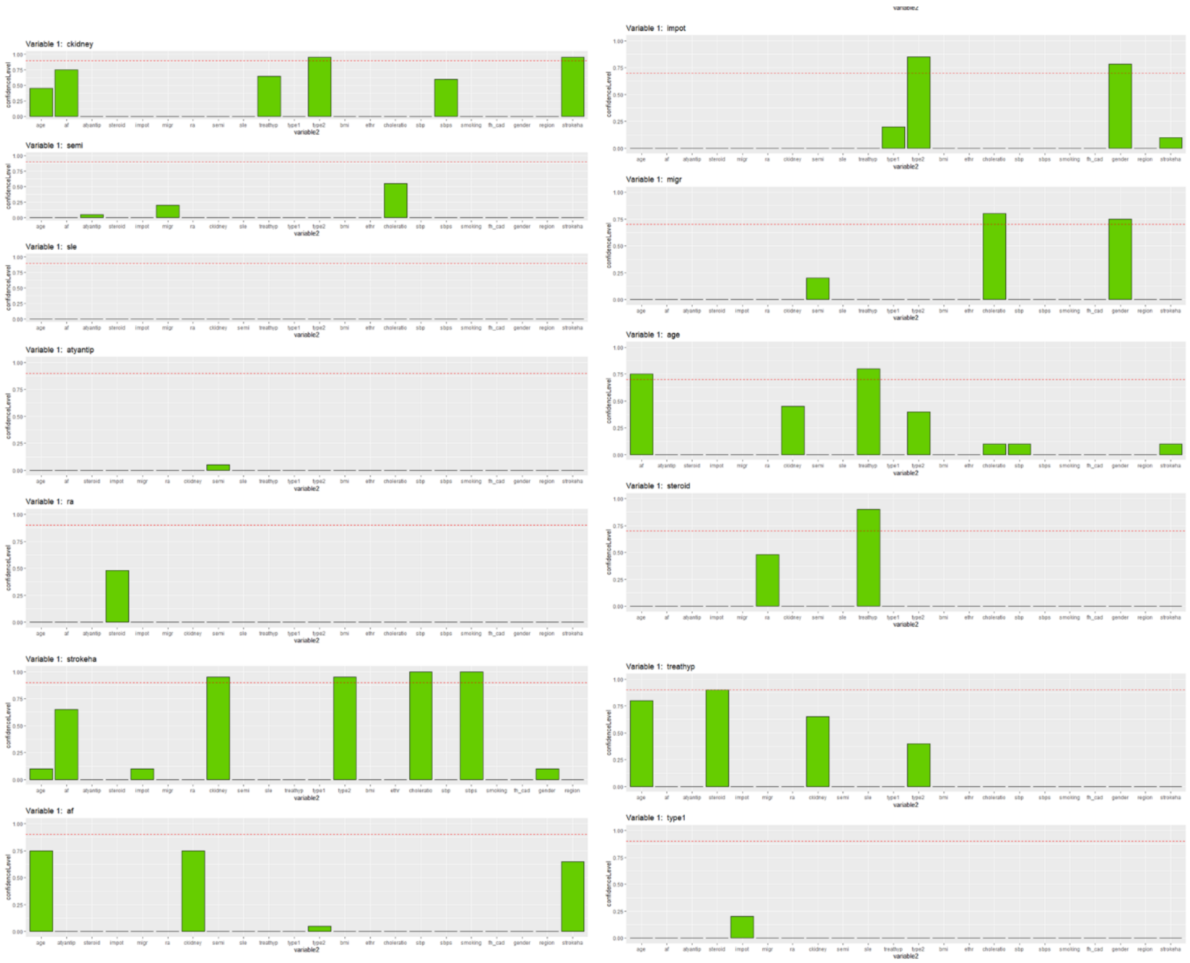

Supplementary Figure 2. Sample Bayesian Network with Latent Variables Included Generated from Resampling the Ground Truth Data and using the FCI algorithm

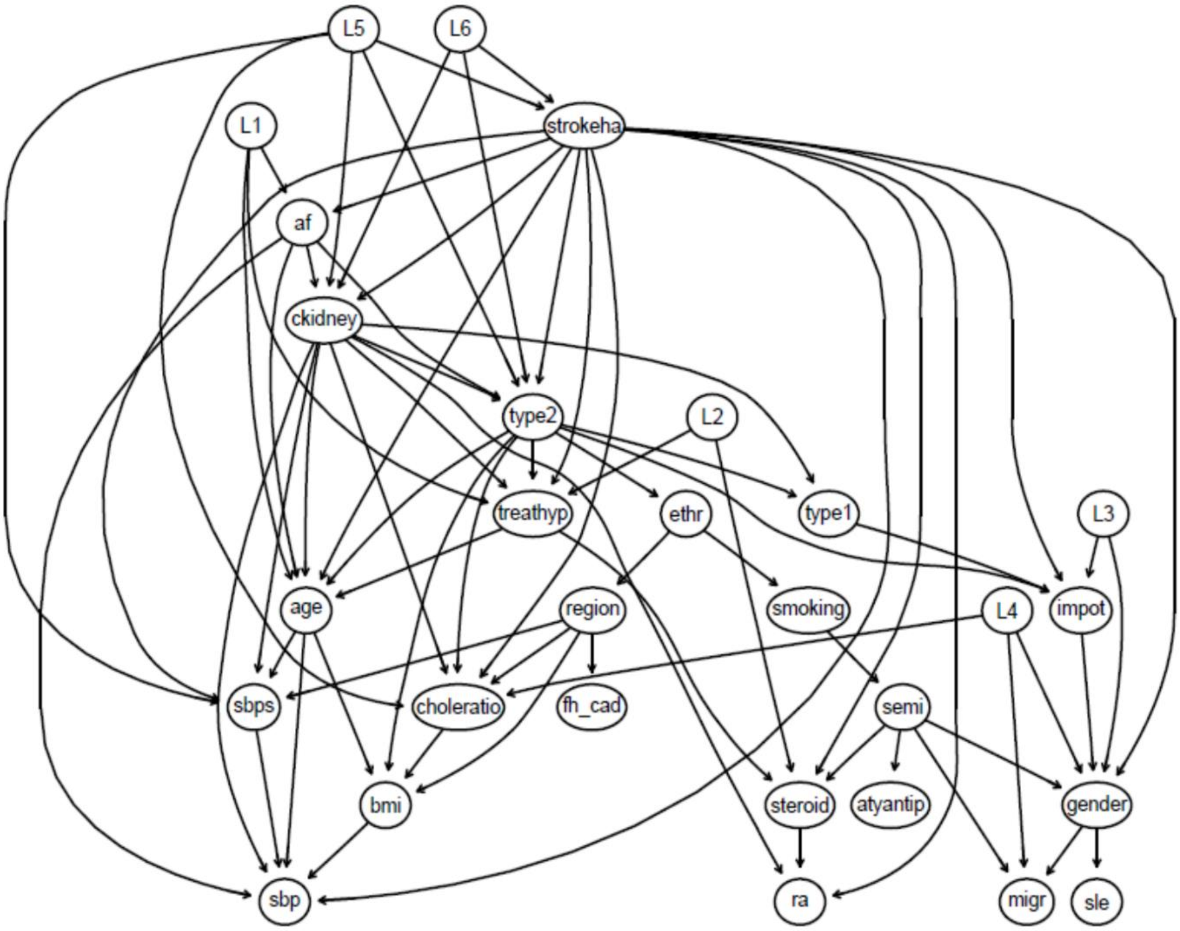

Supplementary Figure 3. AUC analysis for Stepwise Regression showing the ROC and PR curves for Ground Truth (Blue) and Synthetic Data (Red) for increasing numbers of positive and negative cases. Also included are the associated Granger and AUC statistics.

ROC & PR curves (P=Positive Cases, N=Negative Cases)	Granger causality p value (α=0.05): PR, ROC	AUC GT: PR, ROC	AUC SYN: PR, ROC
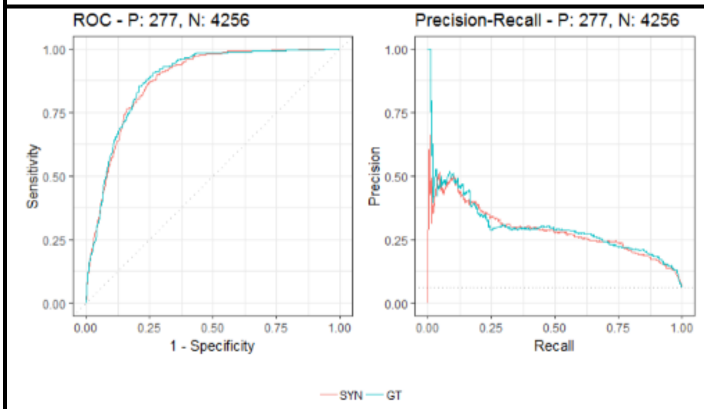	<0.001, 0.358	0.300, 0.881	0.287, 0.877
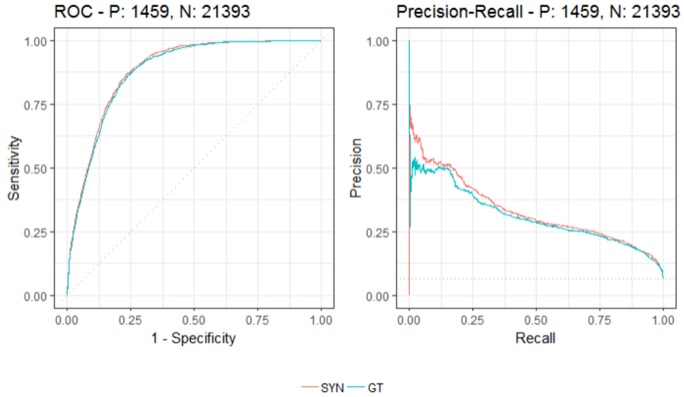	<0.001, <0.001	0.309, 0.878	0.330, 0.884
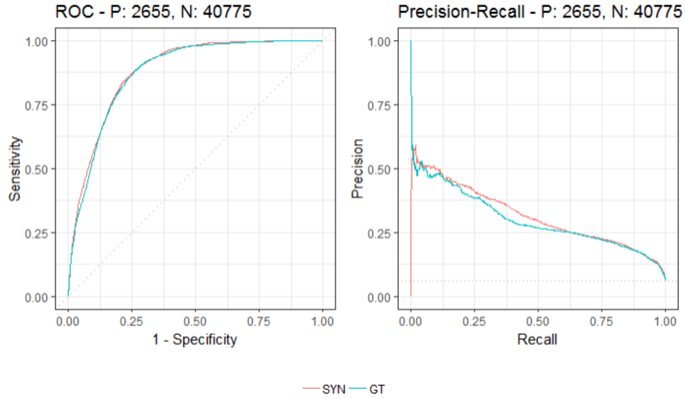	<0.001, <0.001	0.299, 0.876	0.315, 0.882
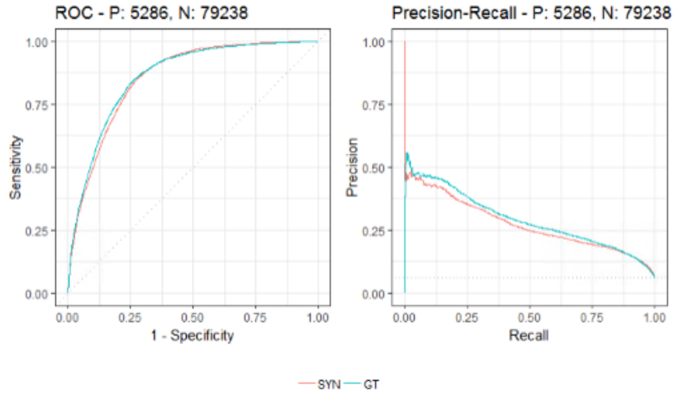	<0.001, <0.001	0.291, 0.860	0.273, 0.855

Supplementary Figure 4. AUC analysis for Linear Discriminant Analysis showing the ROC and PR curves for Ground Truth (Blue) and Synthetic Data (Red) for increasing numbers of positive and negative cases. Also included are the associated Granger and AUC statistics.

ROC & PR curves (P=Positive Cases, N=Negative Cases)	Granger causality p value (α=0.05): PR, ROC	AUC GT: PR, ROC	AUC SYN: PR, ROC
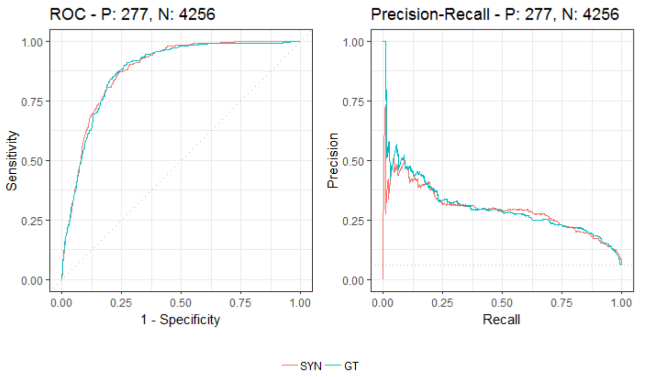	<0.001, <0.001	0.304, 0.878	0.293, 0.881
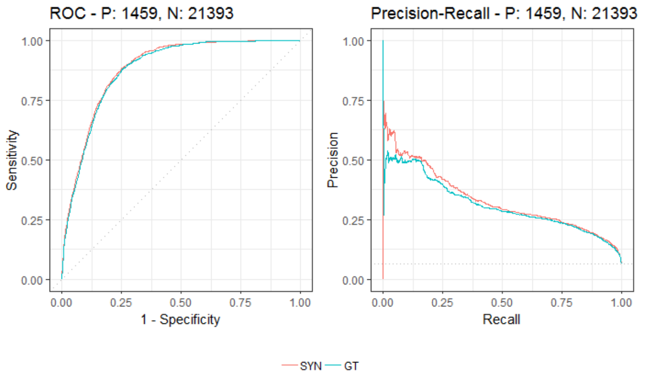	<0.001, <0.001	0.311, 0.879	0.331, 0.884
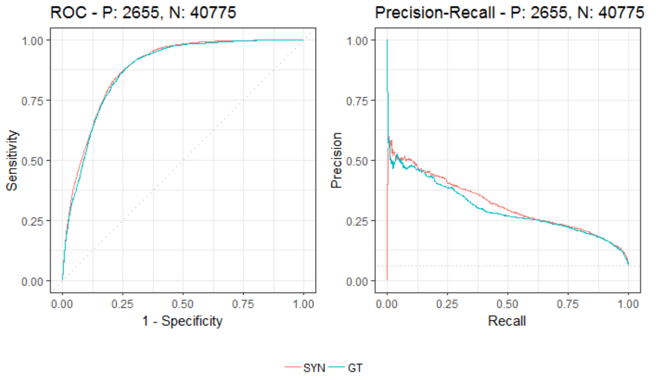	<0.001, <0.001	0.299, 0.876	0.314, 0.882
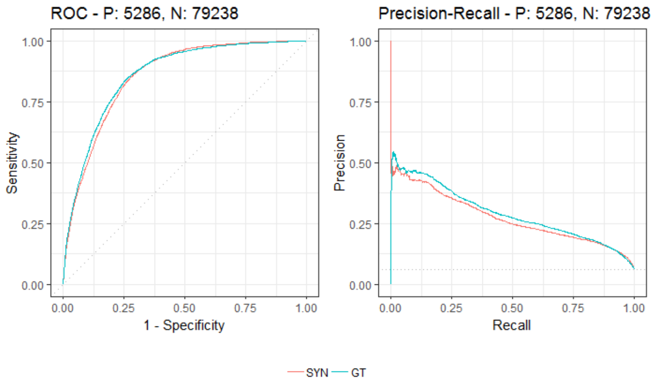	<0.001, <0.001	0.291, 0.860	0.273, 0.855
